# TRICKS Magnetic Resonance Angiography at 3-Tesla for Assessing Whole Lower Extremity Vascular Tree in Patients with High-Grade Critical Limb Ischemia: DSA and TASC II Guidelines Correlations

**DOI:** 10.1100/2012/192150

**Published:** 2012-12-11

**Authors:** Sheung-Fat Ko, Jiunn-Jye Sheu, Chen-Chang Lee, Chung-Cheng Huang, Fan-Yen Lee, Shu-Hang Ng, Yi-Wei Lee, Hon-Kan Yip, Min-Chi Chen

**Affiliations:** ^1^Department of Radiology, Kaohsiung Chang Gung Memorial Hospital and Chang Gung University College of Medicine, 123 Ta-Pei Road, Niao-Sung District, Kaohsiung 833, Taiwan; ^2^Department of Cardiovascular Surgery, Kaohsiung Chang Gung Memorial Hospital and Chang Gung University College of Medicine, 123 Ta-Pei Road, Niao-Sung District, Kaohsiung 833, Taiwan; ^3^Department of Cardiology, Kaohsiung Chang Gung Memorial Hospital and Chang Gung University College of Medicine, 123 Ta-Pei Road, Niao-Sung District, Kaohsiung 833, Taiwan; ^4^Department of Public Health and Biostatistics, Kaohsiung Chang Gung Memorial Hospital and Chang Gung University College of Medicine, 123 Ta-Pei Road, Niao-Sung District, Kaohsiung 833, Taiwan

## Abstract

The entire vascular tree of 58 lower extremities with high-grade critical limb ischemia (CLI) was assessed with three-station time resolved imaging of contrast kinetics (TRICKS) magnetic resonance angiography (T-MRA) and correlated with digital subtraction angiography (DSA) examinations and Trans-Atlantic Inter-Society Consensus II (TASC II) guidelines. Kappa (**κ**) statistics were utilized to evaluate the agreement of stenosis scores (5-point scale; 0 normal to 4 occlusion) based on T-MRA and DSA. With DSA as the standard, significant stenosis instances (stenosis score ≥2) among vascular segments were compared. The **κ**-statistics of image quality (4-point scale; 1 nondiagnostic to 4 excellent) of T-MRA and TASC II classification assessed by a radiologist and a vascular surgeon were also evaluated. Among 870 vascular segments, excellent agreement was observed between T-MRA and DSA (mean **κ** = 0.883) in revealing stenosis (mean stenosis score, 2.1  ±  1.3 versus 2.0  ±  1.3). T-MRA harbored overall high sensitivity (99.5%), specificity (93.6%), positive predictive value (95.4%), negative predictive value (99.6%), and accuracy (97.7%) in depicting significant stenosis. Excellent interobserver agreement (mean **κ** = 0.818) of superb image quality (mean score = 3.5–3.6) of T-MRA and outstanding agreement of TASC II classification of aortoiliac and femoral-popliteal lesions (**κ** = 0.912–0.917) between two raters further verified the clinical feasibility of T-MRA for treatment planning.

## 1. Introduction

Due to its high risk of major tissue loss, critical limb ischemia (CLI) is one of the most severe complications for patients with peripheral arterial occlusive disease (PAOD) [1, 2]. Measurements of the ankle-brachial index (ABI) and duplex ultrasonography are helpful to assess lower limb PAOD; however, they may not provide sufficient anatomic details for treatment planning [1, 3]. Digital subtraction angiography (DSA) is contributive to interventional or preoperative planning, yet it is invasive and uncomfortable [1, 3]. A multidetector row CT with high spatial resolution and long anatomic coverage may evaluate PAOD, but its application is limited by the usage of iodinated contrast medium with a high injection rate, radiation exposure, and obliteration of vascular details caused by calcified plaques [3, 4].

In patients with CLI, contrast-enhanced magnetic resonance angiography (CE-MRA) is a valuable alternative to DSA for treatment planning [1, 3, 5, 6]. However, the great variation of contrast agent travel times and venous contamination in patients with high-grade CLI may hinder the examination of the whole lower extremity with moving table and multiinjection techniques [5, 7]. Time-resolved imaging of contrast kinetics (TRICKS) allows repeated and rapid sampling of signals combined with temporal interpolation to generate time-resolved images from enhanced vessels, so-called TRICKS-MRA (T-MRA). The feasibility of T-MRA at 1.5-tesla for assessing patients with PAOD has been previously advocated in several studies [8–11]. In addition, some researchers have described promising studies of T-MRA focusing in the assessment of infrapopliteal or pedal vessels [12, 13]. InterSociety Consensus for the Management of Peripheral Arterial Disease (TASC II) provided the most important guidelines for the vascular surgeons in the treatment decision of PAOD [1]. At present, three-station T-MRA at 3-tesla for the whole extremity has not been thoroughly addressed and no comparison study between T-MRA at 3-tesla and assessment based on TASC II has been reported. The purpose of this study is to assess the clinical feasibility of three-station three-dimensional (3D) T-MRA at 3-tesla, with low doses for each station, for the evaluation of the entire vascular tree of the lower extremity in patients with high-grade CLI with DSA and TASII correlation.

## 2. Methods

### 2.1. Ethics Statement

Our institutional review board approved this study and waived the need for written informed consent from the participants due to the retrospective and anonymous nature of the analysis.

### 2.2. Patients

Over a 4-year period (January 2006 through January 2010), all patient admitted for management due to high-grade chronic CLI were reviewed. Patients were included based on the following inclusion criteria: (a) high-grade CLI present with ischemic rest pain or ischemic skin lesions, either ulcers or gangrene (Fontaine stage III-IV); (b) featuring a reduced ankle-brachial index (ABI) less than 0.9 at rest and ankle systolic pressure less than 50 mmHg; (c) underwent three-station 3D T-MRA at 3-tesla for assessing the whole vascular tree of the lower extremity; (d) underwent DSA examinations, without or with angioplasty, performed within 7 days (mean, 4 days) after T-MRA; (e) underwent bypass surgery within 3 days (mean, 2 days) after DSA for patients with aortoiliac or femoral-popliteal TASC II type C or type D lesions and/or patients with pedal bypass surgeries due to multivessel and multisegment infrapopliteal PAOD which was inappropriate for angioplasty; (f) clinical followup of at least 2 years (or up to death). Exclusion criteria included (1) the presence of acute limb-threatening ischemia requiring emergency interventional or surgical restoration of blood flow, (2) claustrophobia, (3) cardiac pacemaker insertion, (4) preexisting nephropathy with estimated glomerular filtrations rate (eGFR) <30-mL/min/1.73 m^2^; (5) prior surgeries with prosthesis or metallic clip placements which may induce susceptibility artifact, and (6) comorbidity precluding revascularization. 

### 2.3. Three-Dimensional TRICKS-MRA

All MRA examinations were performed with a 3-tesla MR unit (Signa VH3, GE Medical Systems, Milwaukee, Wis). After well fixation of bilateral lower limbs with elastic foam padding, localizer MR images were obtained using a two-dimensional fast-spoiled gradient-echo sequence in the sagittal plane for each of three stations: lower abdomen to upper thigh, upper thigh to lower knee, and lower knee to foot. Each segment overlapped by at least 5 cm for each station. Three separate 3D T-MRA acquisitions, from the caudal to cranial station, were performed via a body coil. 

There was an 8-minute delay between injections of contrast agents after the leg and feet station, and another after the thigh station. The imaging parameters of T-MRA included: repetition time = 3.6 ms; echo time = 1.6 ms; flip angle = 25°; field of view = 480-mm; 256 × 160 matrix; number of excitations = 0.75; slice thickness = 3.2-mm; 512 × 512 zero-filling reconstruction; temporal resolution = 26 time frames in 4 s; total scan time = 104 s with a mask time of 14 s prior to contrast agent injection. Gadopentate dimeglumine (Magnevist, Bayer-Schering Pharma, Berlin, Germany) was administered using an MR-compatible power injector (Spectris; Medrad, Indianola, PA) via an antecubital vein. Its injection rate was 0.8-mL/s; each injection was followed by 30-mL saline flushing. A total contrast agent dose of 0.17-mmol/kg of body weight was administered at three separate injections (0.07-mmol/kg for legs and feet, 0.05-mmol/kg for thighs, and 0.05-mmol/kg for pelvis) but limit the maximal dose to 20-mL for patient with an eGFR of 30–59 mL/min/1.73 m^2^.

After examination, the raw data were immediately transferred to a commercially available workstation (Advantage Workstation, AW 4.2, GE Healthcare, Milwaukee, WI). The unenhanced mask sequence was automatically subtracted from the subsequent enhanced sequence and mask-subtracted data were reconstructed with the creation of T-MRA along a coronal plane using the maximum-intensity-projection (MIP) algorithm. T-MRA for each station could be displayed in dynamic cine mode. The principle investigator determined the peak arterial time frame(s) of all three stations. The target peak arterial time frame(s) of the aortoiliac, right and left femoral-popliteal and infrapopliteal regions were each further reconstructed separately into 18 images (20° interval) in MIP format so that the whole vascularity tree of the pelvis and each lower limbs could be assessed from various perspectives to avoid overlapping. If necessary, data segmentation was performed to remove unnecessary overlapping structures so that the regions of interest were clearly visible. Finally, all images were transferred to the PACS workstation (Centricity Workstation, version 3.1, GE Healthcare, Milwaukee, WI) equipped with electronic tools for measurement and image review. 

### 2.4. Digital Subtraction Angiography

An experienced radiologist performed all DSA examinations on a digital subtraction system (Integris V5000, Philips Medical Systems, Best, the Netherlands). After insertion of a 5-Fr sheath (or 6 Fr-sheath if angioplasty was planned) via the common femoral artery contralateral to the lower limb with CLI, a 4-Fr pigtail angiographic catheter was inserted into the lower abdominal aorta for aortoiliac DSA. Then selective DSA was performed from the thigh to the foot. Depending on the arterial flow, appropriate injections at a rate of 4–7-mL/s via a power injector were applied at each station. For the general procedure, a total of 40–60-mL of nonionic iodinated contrast agent (iohexol, Omnipaque 300; GE Healthcare Canada Inc, Mississauga, Ontario) was used. In order to improve vascular opacification, selective catheterization was performed whenever possible, however no vasodilative drugs were administered during the DSA procedure. For femoral-popliteal DSA, the catheter tip was placed at the middle part of the external iliac artery when there was no significant aortoiliac stenosis. For infrapopliteal, and pedal DSA, the catheter tip was placed at the middle part of the superficial femoral artery where there was no significant femoral-popliteal stenosis. For all patients, DSA with anteroposterior projection for the evaluation of the aortoiliac, and femoral-popliteal arteries, and anteroposterior and lateral projections for the evaluation of the calf and pedal vessels were obtained. Additional oblique examinations were occasionally performed. In patients with aortoiliac or femoral-popliteal TASC II type A or type B lesions and patients with infrapopliteal lesions appropriate for angioplasty, angioplasty was accomplished during the same session. After the procedure, all DSA images were transferred to the PACS workstation for image review. 

### 2.5. Image Analysis

For evaluation, the extremity considered for treatment was divided into 4 main portions (aortoiliac, femoral-popliteal, infrapopliteal and pedal) and 15 vascular segments (lower abdominal aorta, common iliac, external iliac and common femoral arteries in aortoiliac portion; upper half and lower half of superficial femoral and popliteal arteries in femoral-popliteal portion; upper half and lower half of anterior and posterior tibial and peroneal arteries in infrapopliteal portion; dorsalis pedis and plantar arteries in pedal portion). A radiologist and a vascular surgeon jointly reviewed the T-MRA images on a high-resolution monitor (Barco view, MGD 521MK II, Kortrijk, Belgium) via the PACS system. The degree of stenosis for each vascular segment was assessed by consensus using a 5-point scoring system: 0, indicating normal; 1, minimal stenosis of less than 50%; 2, one lesion with 50% or greater stenosis; 3, more than one lesion with 50% or greater stenosis; 4, occlusion. One month after completion of T-MRA evaluation, the patient order was randomized and DSA images were reviewed using the same method without the T-MRA results. The number of significant stenosis (stenosis score ≥2) for each vascular segment demonstrated on T-MRA and DSA was determined.

The overall quality (including the presence or absence of motion artifact, the continuity of the vessels, and the sharpness and demarcation of the vascular outline) and clinical value of T-MRA in each vascular portion from aortoiliac to pedal level were subjectively graded from a 4-point scale as follows: 1, indicated poor quality, marked venous overlap, and inadequate information for making decisions about further treatment; 2, fair quality, moderate venous overlap, and inadequate information for making decisions about further treatment; 3, good quality, mild or no venous overlap, and adequate information for making decisions about further treatment; and 4, excellent quality, no venous overlap, and adequate information for making decisions about further treatment. Vascular lesions in aortoiliac and femoral-popliteal portions demonstrated on the T-MRA were specifically stratified according to the Trans-Atlantic InterSociety Consensus II (TASC II) guidelines and classification [1]. Type A lesions represent those which yield excellent results from, and should be treated by, endovascular means; type B lesions offer sufficiently good results with endovascular methods that this approach is still principally preferred, unless an open revascularization is required for other associated lesions in the same anatomic area; type C lesions produce superior long-term results with open revascularization that endovascular methods should be employed only for patients at high risk of open repair; and type D lesions do not yield good enough results via endovascular methods to justify primary treatment. The T-MRA images were rated by a vascular surgeon and then independently rated by a radiologist who did not perform the image processing. 

### 2.6. Statistical Analysis

Kappa (*κ*) statistics were used to evaluate the stenosis scores for each vascular segment between T-MRI and DSA (or surgical findings for pedal vessels). The correlation between T-MRI and DSA was considered poor agreement when *κ* < 0.2, fair agreement when *κ* was ≥0.2 to <0.4, moderate agreement when *κ* was ≥0.4 to <0.6, good agreement when *κ* was ≥0.6 to <0.8, and excellent agreement when *κ* was ≥0.8 to 1.0. For the aortoiliac, femoral-popliteal, and infrapopliteal portions, using DSA as the reference standard, the sensitivity, specificity, positive predictive value (PPV), negative predictive value (NPV), and accuracy of T-MRA in the depiction of significant stenosis (stenosis score ≥2) for each vascular segment were, respectively, calculated and compared (Wilcoxon-signed rank test). However, in the pedal portion, surgical findings were regarded as the gold standard when discordance was present between T-MRA and DSA. The Interobserver agreements of the image quality scores for each portion of the lower limb vascular tree and TASC II classification of aortoiliac and femoral-popliteal vascular lesions were also evaluated with *κ* statistics (see Table 2). 

## 3. Results

**Figure 1:**
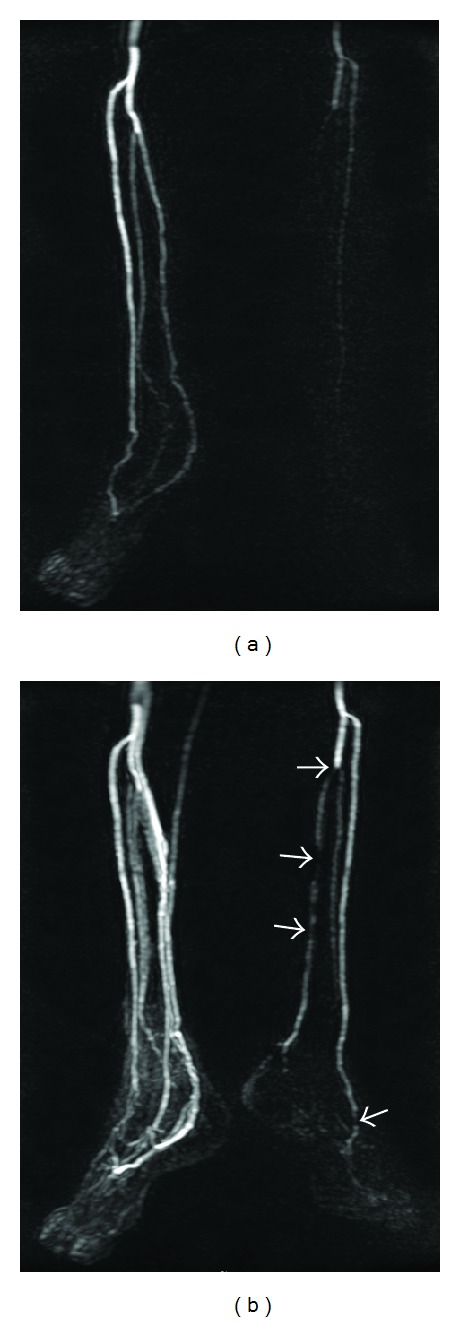
A 60-year-old man with unhealed ulcers over the left heel region for 4 months. (a) T-MRA with MIP reconstruction at 36 s after contrast medium injection demonstrating peak arterial enhancement of the right distal run-off station with clear depiction of infrapopliteal and pedal arteries without venous overlay. The left leg vessels are not poorly opacified. (b) T-MRA at 42 s time frame demonstrating peak arterial enhancement of the left leg with multiple significant stenoses (arrows) in the left posterior tibial artery and dorsalis pedis and nonopacification of planter artery. The right leg is obscured due to prominent venous contamination. Without a time-resolved approach, clear depiction of infrapopliteal and pedal vessels would have been difficult due to discrepant arterial inflow.

The study sample included a total of 58 lower limbs in 55 patients (mean age, 61.2 years; age range, 50–83 years; 21 women and 34 men; Fontaine stage III in 14 patients and IV in 41) who fulfilled the research criteria. In total, diabetes mellitus was noted in 85% of the patients, hypertension in 75%, hypercholesterolemia in 56%, and at least one significant coronary artery stenosis in 39%. A total of 870 segments were ultimately assessed. For 20 patients, obvious discrepant arterial inflow time (>5 sec) to infrapopliteal and pedal portions due to different severity of PAODS between two lower limbs was noted. Discrepant early venous opacification due to lower limb gangrene or inflammation was observed in 28 lower limbs (Figure 1). 

### 3.1. Stenosis Grading and Depiction of Significant Stenosis of the Entire Vascular Tree

**Figure 2:**
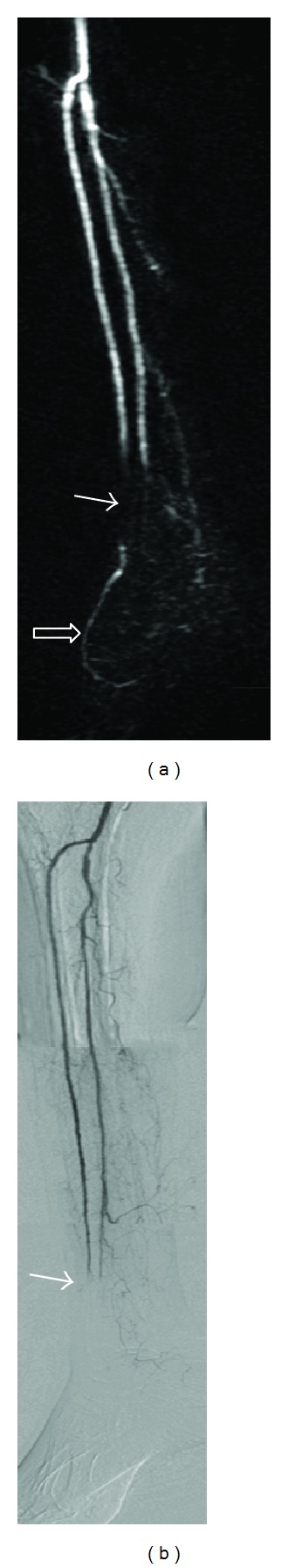
A 67-year-old male with ischemic rest pain and poor healing of the right foot. (a) Targeted peak arterial time frame of T-MRA of the right distal runoff station in oblique reconstruction demonstrating occlusion of posterior tibial artery and distal third of the anterior tibial and peroneal arteries (arrow). Plantar artery is not visible while dorsalis pedis is patent (open arrow). (b) Corresponding DSA demonstrating similar findings to the infrapopliteal portion. The pedal vessels are not opacified. Subsequent surgery confirmed patency of dorsalis pedis artery as demonstrated on T-MRA.

**Figure 3:**
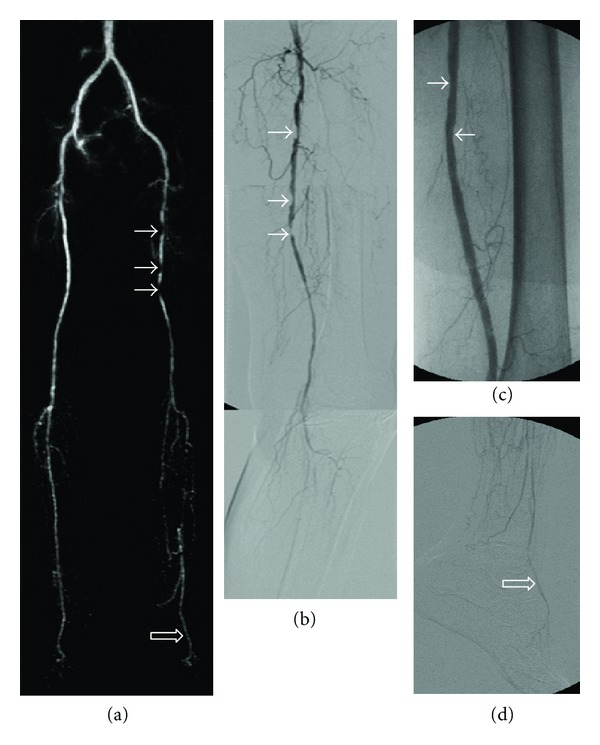
A 72-year-old male with bilateral lower leg ischemic rest pain and left foot ulcers. (a) Concatenated three-station T-MRA demonstrating multiple stenoses (arrows) (type B lesion based on TASC classification) in the left superficial femoral artery and multiple long segmental severe stenoses and occlusions of the infrapopliteal arteries. The left dorsalis pedis artery (open arrow) is patent. (b) Corresponding concatenated DSA demonstrating multiple stenoses in the left superficial femoral artery. The infrapopliteal and pedal arteries beyond the left knee are not opacified. (c) Follow-up angiogram after angioplasty of the left superficial femoral artery showing residual mild stenoses (arrows). (d) Selective DSA of the left foot after angioplasty with improved arterial inflow confirming the patency of the dorsal pedis artery (open arrow); the patient was subsequently managed with popliteal-dorsalis pedis bypass grafting.

Table 1 summarizes the stenosis grading and number of significant stenosis of the entire vascular tree on T-MRA and DSA in 58 lower extremities. The stenosis scores acquired from T-MRA versus DSA at aortoiliac portion (0.8–1.3 versus 0.7–1.2 (mean score), *κ* = 0.787–0.860), femoral-popliteal portion (1.6–2.3 versus 1.6–2.2, *κ* = 0.887–0.929), infrapopliteal portion (2.4–2.8 versus 2.3–2.8, *κ* = 0.857–0.906), and pedal portion (1.8–2.8 versus 1.9–3.0, *κ* = 0.802–0.883) were excellently correlated. In using DSA as the standard, the number of significant stenosis in various segments in aortoiliac, femoral-popliteal, and infrapopliteal portions detected on T-MRA displayed no significant differences from those of DSA (all *P* > .05). Moreover, the sensitivity, specificity, PPV, NPV, and accuracy, respectively, were 96.0–100%, 82.4–100%, 87.5–100%, 96.9–100%, and 94.8–100%. In the pedal portion, four dorsalis pedis and 2 plantar arteries were not opacified on DSA. However, they seemed patent on T-MRA whilst subsequent surgery confirmed vessel patency that enabled the successful creation of popliteal-pedal bypasses (Figure 2). The sensitivity, specificity, PPV, NPV, and accuracy of T-MRA in depicting significant stenosis of pedal vessels were 96.0–100%, 93.9–95.8%, 92.3–97.1%, 96.9–100%, and 94.8–98.3%, respectively. Overall, mild overestimations of significant stenosis were noted in T-MRA when compared to those of DSA (32.1 versus 30.9 (mean number of lesions with stenosis score ≥2)). Among 870 vascular segments, the infrapopliteal segments exhibited the highest stenosis scores and highest numbers of significant stenosis. Overall, there was excellent agreement (*κ* = 0.883) between T-MRA and DSA in revealing stenosis (stenosis score, 2.1 ± 1.3 versus 2.0 ± 1.3) and high-averaged sensitivity (99.5%), specificity (93.6%), PPV (95.4%), NPV (99.6%), and accuracy (97.7%) of T-MRA in revealing significant stenosis of the whole vascular tree (Figure 3).

### 3.2. Correlation of Image Quality and TASC II Classification between Radiologist and Surgeon

 All vascular portions were considered diagnostic on T-MRA with high scores (mean score = 3.5-3.6). Although pedal vessels were less well delineated on T-MRA (mean score = 3.2-3.3), it nonetheless offered important insight for six patients with patent distal arteries that had not been visualized on DSA. Good-to-excellent Interobserver agreement (*κ* = 0.763–0.890) between 2 raters in the validation of T-MRA assessment of the whole vascular tree of the lower extremity were obtained. Excellent agreement (*κ* = 0.912–0.917) between the radiologist and the vascular surgeon in the evaluation of aortoiliac and femoral-popliteal diseases based on TASC II classification were achieved, further verifying T-MRA's treatment planning feasibility.

### 3.3. Clinical Outcome

Among the 58 treated lower extremities, eight were managed with endovascular therapy only, 40 with both endovascular therapy (at least one portion, mostly the femoral-popliteal portion) and surgical bypass grafting (at least one bypass graft, mostly popliteal-pedal type), and 10 with surgical treatment only. The successful limb salvage rate was 100% in 30 days, 96.5% in 1 year, and 94% in 2 years (follow-up duration, 312–1258 days; mean 762 days). None of our patients had any contrast medium-related adverse reaction. During the follow-up period, five patients passed away due to myocardial infarction or pneumonia with sepsis.

## 4. Discussion

 The goal of CLI treatment is the preservation of the affected limb. In addition to maintenance of the quality of life, the cost of patient care following amputation was almost twice that of a successful limb salvage [1, 14]. However, to achieve optimal treatment, revascularization relies on the unimpeded visualization of anatomic details of the entire vascular tree, stenosis, and target vessel for bypass graft [1–3]. Despite high sensitivities and specificities for the detection of significant stenosis or occlusion that have been reported, CE-MRA using bolus-chase and moving table techniques carry inherent limitations for synchronizing the arrival of the contrast agent bolus at the targeted level [1, 9, 15]. As seen half of our patients with high-grade CLI, discrepant arterial inflow due to different PAOD severities between the two lower limbs hampers accurate timing of bolus arrival at different stations [7, 8]. Furthermore, gangrenous and inflammatory changes may result in abnormal vascularities and rapid venous backflows, substantially impacting arterial assessments [6, 9, 16].

MRA with TRICKS technique enables repeat sampling of the critical central *κ*-space during the arrival of contrast bolus in the target vessels combined with temporal interpolation, producing a series of time-resolved images so that the pure arterial phase can be obtained without consideration of injection time [8–13, 17–19]. For CLI patients with small peripheral vessels, high spatial resolution is mandatory to provide details about the leg and pedal vessels for treatment planning. However, at 1.5-tesla, spatial resolution with T-MRA of the infrapopliteal and pedal vessels is limited [17, 18]. At 3-tesla, the signal intensity is fourfold and the signal-to-noise ratio is twofold those of the 1.5-tesla system and thus promotes spatial resolution, improves lesion visibility, shortens acquisition time, and reduces motion artifacts [5, 17, 20]. Furthermore, advances in data acquisition techniques and advanced *κ*-space filling schemes enable the completion of a whole 3D data set in a short duration allowing 3D assessment of target vessels [5, 13, 17, 18, 21]. 

As seen among our cases, with appropriate reconstruction of peak arterial frames of T-MRA of the pelvis and each portion of the lower limbs, venous contamination could be completely eliminated. In addition, the 3D-reconstructed images offer freely-rotated projection angiograms to display vascular lesions from the appropriate perspective avoiding unnecessary overlapping structures. Our results show excellent agreement between T-MRA and DSA (mean *κ* = 0.883) in revealing different degrees of stenoses among the 870 vascular segments. In addition, the present study also reveals excellent diagnostic capability of T-MTA in assessing significant stenosis for different portions of the lower limbs with an overall high sensitivity (99.5%), specificity (93.6%), PPV (95.4%), NPV (99.6%), and accuracy (97.7%). Concurrent with prior reports that suboptimal filling of distal vessels in DSA could lead to nonopacification of patent pedal arteries [9–12], six of our cases with target pedal arteries not visualized on DSA were depicted on T-MRA, enabling the subsequent successful surgical bypass grafting. This study confirms that the 3D three-station T-MRA at 3-tesla is a robust and an accurate method for assessing the entire vascular tree of patients with CLI, even though 75% of our patients had Fontaine stage IV lesions. 

In clinical practice, multiple-injection with high-contrast agent dose protocols (0.3 mmol/kg of body weight) are commonly employed for lower extremity CE-MRA at 1.5-tesla [5–11, 16, 22]. At 3-tesla, noticeable increases in signal-to-noise ratios and blood pool-to-background contrast-to-noise ratio can heighten vascular enhancements permitting contrast agent dose reductions. In addition to the lower cost of the contrast agent, dose-dependent adverse reactions can also be reduced [13, 18, 21]. For instance, the contrast agent dose for 3.0-tesla lower extremity MR angiography can be reduced multifold without compromising image quality [13, 20, 21]. Furthermore, the MRA images at lower contrast doses had better arterial definition than high-dose images, presumably due to lower residual background signaling from the initial contrast injection [9, 21, 23, 24]. Venous contamination was also less frequently observed in the low-dose group [21]. The present study employed low-dose regimen of T-MRA for each station with a total intermediate dose (0.17 mmol/kg) for assessing the entire vascular tree of the lower limb. Even with a low dose for each station, especially coupled with 3D display, this study achieved good anatomic delineation of arterial details with superb image quality (mean score = 3.5-3.6) and excellent Interobserver agreement between two independent raters (mean *κ* = 0.818). 

 For quality treatment planning, TASC II guidelines recommend a multidisciplinary approach for managing CLI patients [1]. The present study also confirms excellent agreement of TASC II classification of aortoiliac and femoral-popliteal diseases (*κ* = 0.912–0.917) between the radiologist and vascular surgeon, further verifying the clinical feasibility of 3D three-station T-MRA. Of note, with optimal planning based on T-MRA findings, even for high-grade CLI as seen among our patients, revascularization with endovascular therapies and/or surgical bypass grafting results in high limb salvage rates even during the 2-year follow-up period. To our knowledge, this is the first large series study of T-MRA at 3-tesla as the primary tool for treatment planning and TASC II assessment in the evaluation of the whole lower extremity vascular tree in patient with high-grade CLI. 

Nevertheless, this study has several limitations. First, we did not compare 3D three-station T-MRA with other CE-MRA techniques. Second, we did not compare the effect of different contrast agent dosages in each station. From a practical point of view, such comparisons may require a high cost of examination and may not be ethical for administering gadolinium-based contrast agent (GBCA) carries the risk of an adverse reaction [25, 26]. On the other hand, in the present study, patients with preexisting nephropathy with GFR <30 mL/min/1.73 m^2^ were excluded and a restricted maximal dose to 20 mL for patient with an eGFR of 30–59 mL/min/1.73 m^2^ was adopted. Such a study protocol could meet the requirements of restrictive GBCA guidelines [25] and the classic labeling of the GBCAs approved by the Food and Drug Administration to decrease the occurrence of nephrogenic systemic fibrosis [26]. Above all, none of our patients had any GBCA-related complications. Third, background subtraction is essential for T-MRA but 3-tesla system is susceptible to motion artifacts. Therefore, comfortable fixation to avoid painfulness of the lower limbs is crucial to examination. Occasionally, mild sedation to minimize rest pain during the procedure could mitigate such effects. Fourth, this study excluded patients with prosthesis or prior surgery with metallic clips and thus was unable to assess the usefulness of T-MRA in such patients. Fifth, T-MTA cannot provide hemodynamic details including the flow velocity and pressure gradient across the stenosis.

In conclusion, for patients with CLI, 3D three-station T-MRA at 3-tesla is clinically feasible for delineating various segments of the entire vascular tree of the lower extremity.

## Figures and Tables

**Table 1:** Comparison of stenosis scores on T-MRA and DSA and depiction of significant stenosis in various segments of the lower extremity.

	Stenosis score			Depiction of significant stenosis						
	T-MRA (mean ± SD)	DSA (mean ± SD)	κ*	T-MRA (*n*)	DSA (*n*)	Sen. %	Spe. %	PPV %	NPV %	Acc. %
Aortoiliac										
Lower aorta	0.8 ± 0.4	0.7 ± 0.5	0.787	1	1	100	100	100	100	100
CIA	1.2 ± 1.0	1.1 ± 1.0	0.875	17	16	100	97.6	94.1	100	98.3
EIA	1.3 ± 1.1	1.2 ± 1.0	0.860	14	13	100	97.8	92.9	100	98.3
CFA	0.8 ± 0.8	0.8 ± 0.8	0.885	8	7	100	98.0	87.5	100	98.3
Femoral popliteal										
USFA	2.2 ± 1.2	2.1 ± 1.2	0.887	41	40	100	94.4	97.6	100	98.3
LSFA	2.3 ± 1.1	2.2 ± 1.0	0.929	44	43	100	93.3	97.7	100	98.3
Popliteal	1.6 ± 1.4	1.6 ± 1.4	0.889	26	25	96.0	93.9	92.3	96.9	94.8
Infrapopliteal										
UATA	2.7 ± 1.2	2.6 ± 1.2	0.857	44	43	100	93.3	97.7	100	98.3
LATA	2.8 ± 1.2	2.8 ± 1.2	0.902	49	48	100	90.0	98.0	100	98.3
UPTA	2.8 ± 1.1	2.8 ± 1.2	0.902	46	44	100	85.7	95.7	100	96.6
LPTA	2.8 ± 1.3	2.7 ± 1.3	0.906	45	44	100	92.9	97.8	100	98.3
UPe	2.4 ± 1.2	2.3 ± 1.1	0.840	41	40	100	94.4	97.6	100	98.3
LPe	2.5 ± 1.1	2.5 ± 1.2	0.862	44	41	100	82.4	93.2	100	94.8
Pedal**										
DP	1.8 ± 1.2	1.9 ± 1.3	0.812	26	25**	96.0	93.9	92.3	96.9	94.8
PL	2.8 ± 1.2	3.0 ± 1.3	0.802	35	34**	100	95.8	97.1	100	98.3

Average	2.1 ± 1.3	2.0 ± 1.3	0.883	32.1	30.9	99.5	93.6	95.4	99.6	97.7

T-MRA: TRICKS-magnetic resonance angiography; DSA: digital subtraction angiography; (*n*): number of patients with at least one significant stenosis; Sen.: sensitivity; Spe.: specificity; NPV: negative predictive value; PPV: positive predictive value; Acc.: accuracy; CIA: common iliac artery; EIA: external iliac artery, CFA: common femoral artery; USFA and LSFA: upper and lower superficial femoral artery; UATA and LATA: upper and lower anterior tibial artery; UPTA and LPTA: upper and lower posterior tibial artery, UPe and LPe: upper and lower peroneal artery; DP: dorsal pedis; PL: plantar artery

**Surgical findings was regarded as gold standard while there were discordance between T-MRA and DSA (4 DP and 2PL were not opacified on DSA but seemed patent on MRA with subsequent surgical confirmation of patency). *Stenosis scores (T-MRA versus DSA) were assessed with kappa statistics.

**Table 2:** Agreement of quality scores and TASC II classification of T-MRA between the radiologist and vascular surgeon.

	Quality score			TASC II classification		
	Radiologist	Surgeon	κ*	Radiologist	Surgeon	κ*
	(mean ± SD)	(mean ± SD)				
Aortoiliac	3.7 ± 0.6	3.6 ± 0.6	0.804	A (*n* = 14)	A (*n* = 14)	0.917
				B (*n* = 4)	B (*n* = 4)	
				C (*n* = 2)	C (*n* = 1)	
				D (*n* = 2)	D (*n* = 3)	
Femoral popliteal	3.8 ± 0.4	3.8 ± 0.4	0.890	A (*n* = 27)	A (*n* = 27)	0.912
				B (*n* = 10)	B (*n* = 10)	
				C (*n* = 4)	C (*n* = 3)	
				D (*n* = 12)	D (*n* = 13)	
Infrapopliteal	3.7 ± 0.5	3.7 ± 0.5	0.763	NA	NA	
Pedal	3.2 ± 0.6	3.3 ± 0.5	0.791	NA	NA	

Average	3.6 ± 0.6	3.5 ± 0.5	0.818			

T-MRA: TRICKS-magnetic resonance angiography; DSA: digital subtraction angiography; NA: not applicable.

*Quality scores and TASC II classification (radiologist versus surgeon) were assessed with kappa statistics.
